# *Lactobacillus plantarum* Lipoteichoic Acids Possess Strain-Specific Regulatory Effects on the Biofilm Formation of Dental Pathogenic Bacteria

**DOI:** 10.3389/fmicb.2021.758161

**Published:** 2021-11-15

**Authors:** Dongwook Lee, Jintaek Im, Dong Hyun Park, Sungho Jeong, Miri Park, Seokmin Yoon, Jaewoong Park, Seung Hyun Han

**Affiliations:** ^1^Department of Oral Microbiology and Immunology, and DRI, School of Dentistry, Seoul National University, Seoul, South Korea; ^2^Bio Research Team, Lotte R&D Center, Seoul, South Korea

**Keywords:** biofilm, *Lactobacillus plantarum*, lipoteichoic acid, dental pathogens, human dentin slice

## Abstract

Bacterial biofilm residing in the oral cavity is closely related to the initiation and persistence of various dental diseases. Previously, we reported the anti-biofilm activity of *Lactobacillus plantarum* lipoteichoic acid (Lp.LTA) on a representative dental cariogenic pathogen, *Streptococcus mutans*. Since LTA structure varies in a bacterial strain-specific manner, LTAs from various *L. plantarum* strains may have differential anti-biofilm activity due to their distinct molecular structures. In the present study, we isolated Lp.LTAs from four different strains of *L. plantarum* (LRCC 5193, 5194, 5195, and 5310) and compared their anti-biofilm effects on the dental pathogens, including *S. mutans*, *Enterococcus faecalis*, and *Streptococcus gordonii*. All Lp.LTAs similarly inhibited *E. faecalis* biofilm formation in a dose-dependent manner. However, their effects on *S. gordonii* and *S. mutans* biofilm formation were different: LRCC 5310 Lp.LTA most effectively suppressed the biofilm formation of all strains of dental pathogens, while Lp.LTAs from LRCC 5193 and 5194 hardly inhibited or even enhanced the biofilm formation. Furthermore, LRCC 5310 Lp.LTA dramatically reduced the biofilm formation of the dental pathogens on the human dentin slice infection model. Collectively, these results suggest that Lp.LTAs have strain-specific regulatory effects on biofilm formation of dental pathogens and LRCC 5310 Lp.LTA can be used as an effective anti-biofilm agent for the prevention of dental infectious diseases.

## Introduction

Bacterial biofilms are defined as the aggregation of microorganisms attached to a surface of various biotic and abiotic materials (Scharnow et al., 2019). Biofilm formation is initiated from the adherence of planktonic bacteria to these surfaces, and the adhered bacteria subsequently produce an extracellular polymeric substance (EPS) consisting of proteins, polysaccharides, and extracellular DNAs that enfold bacteria within the biofilm (Flemming et al., 2007). Since EPS provides a physical barrier to embedded bacteria, the bacteria in biofilm have greater resistance to antibiotics and anti-microbial treatments than planktonic bacteria (Stewart, 2015) and can easily evade host immune responses, including phagocytosis by macrophages or neutrophils (Thurlow et al., 2011; Domenech et al., 2013). For these reasons, bacterial biofilm is considered as a major public health concern that limits treatment options for bacterial infections.

Among various oral microorganisms, *Streptococcus mutans*, *Enterococcus faecalis,* and *Streptococcus gordonii* are considered as major Gram-positive, facultative anaerobic dental pathogens predominantly found in the oral cavity of patients with apical periodontitis or dental caries (Lee, 2017; Scharnow et al., 2019; Park et al., 2020). These dental pathogens can initiate and/or exacerbate the symptoms of the diseases by biofilm formation cooperating with other cariogenic or periodontopathic bacteria on root canal walls or dentin surfaces (Abranches et al., 2018; Park et al., 2020). In fact, accumulating studies have demonstrated that three-dimensional structure of *E. faecalis*, *S. gordonii*, or *S. mutans* biofilm provides enhanced protection against antibiotics, medical treatments, and host immunity (Loo et al., 2000; Krzysciak et al., 2014; Ch’ng et al., 2019). For instance, *E. faecalis* biofilm shows 1,000-fold more resistance to multiple antibiotics and disinfectants, such as chlorhexidine digluconate and calcium hydroxide, compared to planktonic state of the bacteria (Ghaziasgar et al., 2019). Moreover, previous studies have shown that the currently available dental medicaments have limited effectiveness against biofilm of dental pathogens and their long-term use accompanies various adverse effects, such as taste alteration, soft-tissue lesions, teeth staining, and allergic responses (Bramante et al., 2008; Mohammadi and Abbott, 2009; Najafi et al., 2012). Hence, alternative strategies for controlling biofilm of dental pathogens with marginal adverse effects are urgently demanded to efficiently prevent and/or treat biofilm-related dental diseases.

Recently, natural anti-biofilm agents have been developed as alternatives to overcome the defects of traditional dental medicaments. Previous studies have shown that various *Lactobacillus* species, widely used as probiotic bacteria, can be used as an anti-biofilm agent against dental pathogens without accompanying any adverse effect. In fact, *Lactobacillus plantarum*, *Lactobacillus reuteri*, and *Lactobacillus rhamnosus* GG suppress biofilm formation of *S. mutans* (Soderling et al., 2011), and anti-biofilm activity of *Lactobacillus kefiranofaciens* against *S. mutans* and *Streptococcus sobrinus* has also been reported (Jeong et al., 2018). In addition, *Lactobacillus*-derived molecules with anti-biofilm activity have been extensively studied based on anti-biofilm characteristics of *Lactobacillus*. For instance, *Lactobacillus*-derived biosurfactants suppress biofilm formation of *Candida albicans*, a causative etiological agent of dental caries (Ceresa et al., 2015), and culture supernatant from *L. plantarum* efficiently inhibits *S. mutans* biofilm formation (Ahn et al., 2018a; Wasfi et al., 2018).

Among various *Lactobacillus*-derived molecules, lipoteichoic acid (LTA) is known as a major cell wall component found in Gram-positive bacteria involved in bacterial growth, adhesion, biofilm formation, and stimulation of host immunity (Weidenmaier and Peschel, 2008; Kang et al., 2016). In general, LTA is an amphiphilic glycolipid linked to a hydrophilic polyphosphate polymer, and five structurally distinct types of LTAs (from types I to V) have been identified from various bacterial species (Kang et al., 2016). In the structure, LTA from *L. plantarum* possesses poly-glycerolphosphate (Gro-P) backbone, which is composed of phosphate and glycerol containing D-alanine, glucose, or galactose residues, linked with triacylated glycolipid moiety, while LTA from *Staphylococcus aureus* has Gro-P backbone with N-acetylglucosamine or D-alanine residues linked with diacylated glycolipid anchor (Jang et al., 2011). The structural diversity of LTA seems to be closely related to its biological characteristics. In fact, LTA from *L. plantarum* was less immunostimulatory for inducing pro-inflammatory mediators, such as nitric oxide and tumor necrosis factor-alpha than LTA from *S. aureus* (Ryu et al., 2009). Furthermore, *L. plantarum* LTA appears to possess anti-inflammatory properties. For instance, *L. plantarum* LTA attenuated the IL-8 production induced by a synthetic bacterial lipopeptide, Pam2CSK4, and a synthetic analog of viral double-stranded RNA, poly I:C, in human and porcine intestinal epithelial cells, respectively (Noh et al., 2015; Kim et al., 2017).

In our previous studies, we found that LTAs from *Lactobacillus* species effectively inhibited biofilm formation and/or disrupted the preformed biofilm of diverse pathogens, including *E. faecalis*, *S. mutans*, and *S. aureus* (Ahn et al., 2018a,b; Jung et al., 2019; Kim et al., 2019, 2020). Moreover, among the tested LTAs from *Lactobacillus* species, *L. plantarum* LTA (Lp.LTA) showed the most potent anti-biofilm activity against dental pathogens, such as *E. faecalis* and *S. mutans*, suggesting that LTA structure may determine its anti-biofilm capacity (Ahn et al., 2018a; Jung et al., 2019). However, the regulatory effects of LTAs from different *L. plantarum* strains on biofilm formation of dental pathogens have yet to be evaluated and characterized. Therefore, we evaluated the anti-biofilm activities of LTAs from four different strains of *L. plantarum* to select Lp.LTA having the most effective anti-biofilm activity against clinically-isolated the dental pathogens, including *E. faecalis*, *S. mutans*, and *S. gordonii*.

## Materials and Methods

### Isolation of *L. plantarum* Strains From Kimchi

A total of four strains of *L. plantarum* LRCC 5193, 5194, 5195, and 5310 were isolated from Kimchi as previously described (Kim et al., 2018; Lim et al., 2018). Briefly, homemade and commercial Kimchi samples were collected from local houses (Icheon, Republic of Korea) and markets (Jecheon, Republic of Korea), respectively. The Kimchi samples were homogenized and serially diluted with peptone water (0.85% mass/vol). The diluted samples were then spread on de Man, Rogosa, and Sharpe (MRS; Difco Laboratories, Franklin Lakes, NJ, United States) agar plates. After the incubation at 37°C for 2 or 3days, single colonies having yellow circles were selected and then streaked on MRS agar plates to obtain pure cultures. The 16S rRNA gene sequence analysis was then conducted to identify the bacteria.

### Bacteria and Culture Conditions

For the LTA purification, the bacteria were grown in MRS broth (Difco Laboratories) for 48h at 37°C in a capped bottle without agitation. A total of 12 strains of dental pathogenic bacteria, including four strains each from *E. faecalis*, *S. gordonii,* and *S. mutans*, were used to determine the regulatory effect of Lp.LTAs on their biofilm formation. As shown in Table 1, three laboratory strains of dental pathogenic bacteria were provided by University of California at San Francisco (Prof. Paul Sullam from UCSF, San Francisco, CA), or the American Type Culture Collection (ATCC, Manassas, VA), while nine clinically isolated strains of dental pathogenic bacteria, recovered from dental plaque of patients with periodontitis, gingivitis, or dental caries, were provided by the Korean Collection for Oral Microbiology (KCOM, Gwangju, Republic of Korea). All of the dental pathogenic bacteria were grown in Todd-Hewitt medium (Difco Laboratories) supplement with 1% yeast extract (Difco Laboratories; THY) at 37°C with shaking or static under aerobic conditions.

**Table 1:** Information of dental pathogenic bacteria strains.

Bacteria	Source^a^
*Enterococcus faecalis*	
ATCC 29212	ATCC, laboratory strain
KCOM 1083	KCOM, clinical isolate (dental plaque)
KCOM 1161	KCOM, clinical isolate (dental plaque)
KCOM 5291	KCOM, clinical isolate (dental plaque)
*Streptococcus gordonii*	
CH1	UCSF, laboratory strain
KCOM 1967	KCOM, clinical isolate (dental plaque)
KCOM 2106	KCOM, clinical isolate (dental plaque)
KCOM 2867	KCOM, clinical isolate (dental plaque)
*Streptococcus mutans*	
ATCC 25175	ATCC, laboratory strain
KCOM 1054	KCOM, clinical isolate (dental plaque)
KCOM 1116	KCOM, clinical isolate (dental plaque)
KCOM 1223	KCOM, clinical isolate (dental plaque)

a ATCC, American Type Culture Collection; KCOM, Korean Collection for Oral Microbiology; UCSF, University of California at San Francisco.

### Purification of LTAs

Highly pure and structurally intact LTAs from four strains of *L. plantarum* (LRCC 5193, 5194, 5195, and 5310) were purified as previously described (Ryu et al., 2009). The bacterial pellet of each strain of *L. plantarum* harvested by centrifugation was resuspended in 0.1M sodium citrate buffer (pH 4.7) and disrupted by ultrasonication (Vibracell VCX500; Sonics and materials, Newtown, CT, United States). The bacterial lysates were mixed with an equal volume of *n*-butanol, and the lower phase was collected by centrifugation. The collected lower phase was then dialyzed in a semi-permeable dialysis membrane (Spectrum Laboratories, Rancho Dominguez, CA, United States) against endotoxin-free distilled water (Daihan Pharm. Co. Ltd., Seoul, Republic of Korea). The dialyzed extracts were equilibrated with 15% *n*-propanol in 0.1M sodium acetate buffer and subjected to hydrophobic interaction chromatography using a column filled with octyl-Sepharose (GE healthcare, Chicago, IL, United States). After washing out unbound materials with 15% *n*-propanol in 0.1M sodium acetate buffer, the bound materials were eluted with 35% *n*-propanol in 0.1M sodium acetate buffer in a series of 7ml aliquots using a fraction collector (Bio-Rad, Hercules, CA, United States). According to the result of inorganic phosphate assay, the column fractions containing phosphate, that were considered as LTA-containing elutes, were pooled and dialyzed again under the aforementioned condition. The dialyzed fractions were equilibrated with 30% *n*-propanol in 0.1M sodium acetate buffer and subjected to ion-exchange chromatography using a column filled with DEAE-Sepharose (Sigma-Aldrich, St. Louis, MO, United States) equilibrated in 30% *n*-propanol in 0.1M sodium acetate buffer. A series of 7ml fractions were further eluted with a linear salt gradient (0 to 1M NaCl in the equilibration buffer). Based on the results from inorganic phosphate assay, the column fractions containing phosphate were pooled, dialyzed, and lyophilized. The quantity of LTA was then determined by measuring the dry weight of purified LTA. Possible impurities, such as endotoxins, nucleic acids, and proteins, were examined in the Lp.LTA preparations using *Limulus* amoebocyte lysate assay kit (Lonza, Basel, Switzerland), spectrophotometer (Nano Drop 2000; Thermo Fisher Scientific, Waltham, MA, United States), and BCA protein assay kit (Thermo Fisher Scientific), respectively.

### Inorganic Phosphate Assay

The LTA-containing fractions were identified by inorganic phosphate assay as previously described (Han et al., 2003). Briefly, the elutes from the hydrophobic interaction and ion-exchange chromatography were mixed with sulfuric acid (Junsei, Tokyo, Japan) and nitric acid (Junsei) and boiled. The mixture was then neutralized with 3M sodium hydroxide (Junsei) and mixed with 100mM molybdate in 5M sulfuric acid and 130mM stannous chloride in glycerol. After incubation for 5min, optical density was measured at 600nm using a microplate reader (Molecular Devices, CA, United States). Potassium phosphate (Sigma-Aldrich) was used as standard for phosphate content determination.

### Western Blot Analysis

Western blot analysis for the prepared Lp.LTAs was conducted as previously described (Lee et al., 2019b). Briefly, 0.5 or 2.0μg of each Lp.LTA was subjected to 15% SDS-PAGE and then electro-transferred to a PVDF membrane (Millipore, Bedford, MA, United States) using a tank transfer system (Bio-Rad, Hercules, CA, United States). The membrane was washed with Tris-buffered saline containing Tween 20 (TBST; 20mM Tris-HCl, 150mM NaCl, and 0.05% Tween 20) and incubated in a blocking buffer (5% skim milk in TBST) for 1h. The membrane was incubated with specific primary antibody recognizing poly-Gro-P region of LTA (Hycult, Wayne, PA, United States) at 4°C overnight. After washing with TBST, the membrane was further incubated with HRP-conjugated anti-mouse IgG (Jackson Immuno Research, West Grove, PA, United States), and the immunoreactive bands on the membrane were detected with ECL reagents (Amersham Biosciences, Princeton, NJ, United States). The image was obtained and analyzed using Vilber bio-image analyzer (Vilber Lourmat, Marne-la-Vallée, France).

### Crystal Violet Staining

Biofilm formation of dental pathogenic bacteria was determined by crystal violet staining as previously described (Kim et al., 2020). All strains of *E. faecalis*, *S. gordonii*, and *S. mutans* on 96-well plates (1×10^7^CFU/ml) were grown in THY medium supplemented with 0.06–0.3% glucose (for *E. faecalis* and *S. gordonii*) or 0.01–0.2% sucrose (for *S. mutans*) in the presence or absence of each Lp.LTA at 37°C for 24h. The biofilms were gently washed with phosphate-buffered saline (PBS) and stained with 0.1% crystal violet solution for 20min at room temperature. The stained biofilm was then dissolved in dissociation buffer (95% ethanol and 0.1% acetic acid in distilled water), and optical density at 600nm was measured using a microplate reader (Molecular Devices).

### Colony-Forming Unit

Colony-forming unit (CFU) formation assay was conducted as previously described (Castillo Pedraza et al., 2017) with minor modification. Briefly, biofilms of all strains of *E. faecalis*, *S. gordonii*, and *S. mutans* were formed on 24-well plates (1×10^7^CFU/ml) in the presence or absence of various concentrations of LRCC 5310 Lp.LTA at 37°C for 24h. After washing the biofilm with PBS, it was scraped off and resuspended in 1ml PBS. To titrate bacterial CFU within the biofilm, serially diluted bacterial resuspensions (from 10^−1^ to 10^−5^) were seeded on the THY agar plate and incubated at 37°C for 48h. Colony formations were then examined, and CFUs were calculated based on the dilution fold.

### Confocal Laser Scanning Microscopy

Dental clinical isolates of *E. faecalis* (KCOM 1083), *S. gordonii* (KCOM 1967), and *S. mutans* (KCOM 1223) on confocal glass-bottom dishes (1×10^7^CFU/ml) were grown in THY medium supplemented with 0.3 and 0.03% glucose (for *E. faecalis* KCOM 1083 and *S. gordonii* KCOM 1967) or 0.03% sucrose (for *S. mutans* KCOM 1223) in the presence or absence of 30μg/ml of LRCC 5310 Lp.LTA at 37°C for 24h. The biofilms were gently rinsed with PBS to remove planktonic bacteria and stained with the LIVE/DEAD BacLight Bacterial Viability Kit (Thermo Fisher Scientific) containing SYTO9 and propidium iodide according to the manufacturer’s instructions. After washing with PBS, the stained biofilms were visualized by a confocal laser scanning microscope (LSM 800; Zeiss, Oberkochen, Germany). Simultaneous dual-channel imaging was used to display green (for SYTO9 at 480/500nm) and red fluorescence (for propidium iodide at 490/635nm). The biomass and thickness distribution of biofilms were quantified using COMSTAT2 software (Heydorn et al., 2000).

### Preparation of Human Dentin Slices

Preparation of human dentin slices for experimental purpose was approved by the Institutional Review Board of Seoul National University Dental Hospital, Seoul, Republic of Korea (CRI 17010). Human dentin slices were prepared as previously described (Lee et al., 2019a). Briefly, extracted human single-rooted premolars were cleansed using an ultrasonic scaler before preparing 500μm thick cross sections using Isomet precision saw (Buehler, Lake Bluff, IL, United States). The cross-sectioned dentin slices were sequentially treated with 17% EDTA, 2.5% sodium hypochlorite and 5% sodium thiosulfate for 5min in each buffer. After autoclaving at 121°C for 15min, prepared dentin slices were stored at room temperature until subjected to scanning electron microscopic analysis.

### Scanning Electron Microscopic Analysis

Biofilm formation of dental pathogenic bacteria on human dentin slices in the presence or absence of Lp.LTA was visualized by scanning electron microscopic analysis as previously described (Kim et al., 2019). Briefly, *E. faecalis* (KCOM 1083), *S. gordonii* (KCOM 1967), and *S. mutans* (KCOM 1223) were grown on dentin slices in THY medium supplemented with 0.3 and 0.03% glucose (for *E. faecalis* KCOM 1083 and *S. gordonii* KCOM 1967) or 0.03% sucrose (for *S. mutans* KCOM 1223) in the presence or absence of 30μg/ml of LRCC5310 Lp.LTA at 37°C for 24h. After the incubation, dentin slices were gently rinsed with PBS to remove planktonic bacteria and prefixed with 2.5% glutaraldehyde and 2% paraformaldehyde in PBS at 4°C overnight. The dentin slices were further fixed with 1% osmium tetroxide for 90min and subsequently dehydrated with gradually higher concentrations of ethanol (70, 80, 90, 95, and 100% for 15min in each solution). After drying gold-coated sputter with hexamethyldisilazane, the biofilm formation on dentin slices was examined by a scanning electron microscope (Apreo 2; Thermo Fisher Scientific).

### Statistical Analysis

Values are expressed as mean values±standard deviations from triplicate of each treatment group. Statistical significance between indicated Lp.LTA treatment and non-treatment groups was analyzed with Student’s *t* test. Asterisks (*) indicate experimental groups that are significantly different (*p*<0.05) from the designated control.

## Results

### Lp.LTAs Have Differential Structures Depending on Their Strains

**Figure 1:**
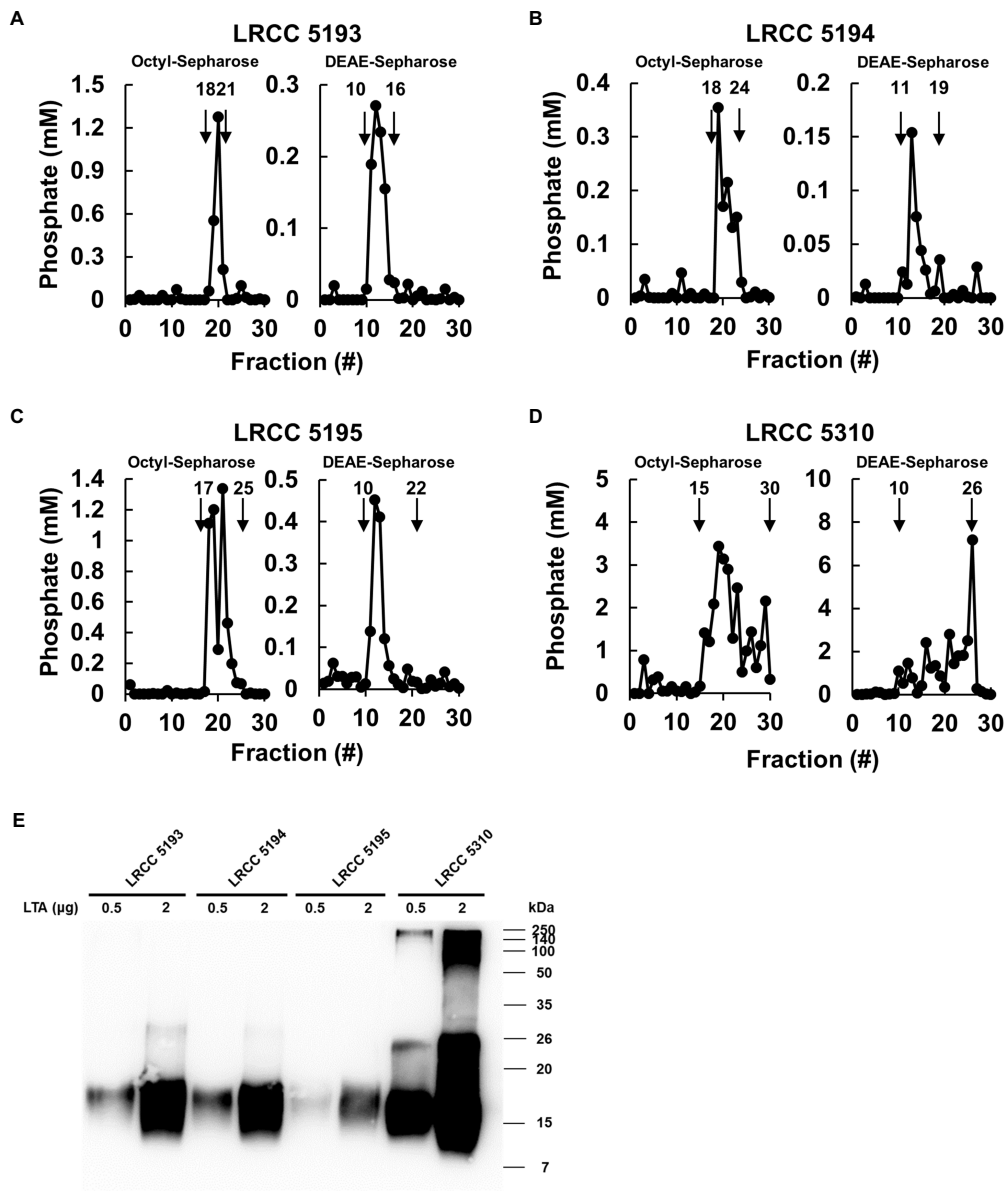
Elution profiles and molecular characteristics of LTAs purified from four different strains of *L. plantarum*. **(A–D)** LTAs were purified from four different strains of *L. plantarum* by sequential application of butanol extraction, hydrophobic interaction chromatography, and ion-exchange chromatography. Inorganic phosphate assays were then performed to measure the quantity of LTAs in each fraction after hydrophobic interaction chromatography (Octyl-Sepharose, left) and ion-exchange chromatography (DEAE-Sepharose, right) as described in Materials and Methods. **(E)** Purified LTAs were subjected to Western blot analysis using antibody specific for LTA.

To examine whether Lp.LTAs have strain-specific anti-biofilm activity on biofilm formation of dental pathogenic bacteria, such as *E. faecalis*, *S. gordonii*, and *S. mutans*, we initially purified LTAs from four different strains of *L. plantarum* (LRCC 5193, 5194, 5195, and 5310) under the same condition. Inorganic phosphate assay was then performed for each column fractions from the hydrophobic interaction and ion-exchange chromatography because phosphate contents of column fractions are linearly correlated with their LTA contents (Morath et al., 2001). Phosphate containing fractions of Lp.LTAs from LRCC 5193, 5194, and 5195 showed relatively similar fractions ranging from 17 to 25 (Figures 1A–C, left), while phosphate was detected in broader column fractions of LRCC 5310 Lp.LTA (15 to 30) following purification by the hydrophobic interaction chromatography (Figure 1D, left). After ion-exchange chromatography, the detected ranges of phosphate fraction numbers of LRCC 5193 (10 to 16) and 5194 (11 to 19) were relatively similar (Figures 1A,B, right). However, ranges of LRCC 5195 (10 to 22) and 5310 (10 to 26) were similar and broader than the previous strains, indicating differential net charge and hydrophobicity of Lp.LTA strains (Figures 1C,D, right). Moreover, since an antibody used for Western blot analysis recognizes the Gro-P backbone of LTA, the different migration of each LTA indicated that *L. plantarum* LRCC 5310 strain contained a broader size of Gro-P backbone than LTAs from other strains (Figure 1E). Collectively, these results suggested a possibility that each *L. plantarum* strain possesses structurally different LTA in net charge, hydrophobicity, and size of its poly-Gro-P backbone on their cell wall. Next, we examined the contents of possible impurities, including endotoxin, protein, and nucleic acid in the isolated LTAs, together with the amount of LTA from each *L. plantarum* strain. As shown in Table 2, small amounts of endotoxin, proteins, and nucleic acids were detected in all of the isolated LTAs. However, since our previous studies demonstrated that these levels of impurities had negligible biological activities (Ryu et al., 2009; Hong et al., 2014), we utilized these Lp.LTAs for examining their effects on biofilm formation of dental pathogens.

**Table 2:** Contents of impurities and recovered LTAs.

LTA	Endotoxin^a^ (EU/mg LTA)	Protein^b^ (ng/mg LTA)	Nucleic acid^c^ (μg/mg LTA)	Amount of LTA recovered^d^ (μg/g bacterial pellet)
LRCC 5193	5.6	4.5	18.3	25
LRCC 5194	9.0	1.4	12.6	18
LRCC 5195	6.4	1.4	12.6	42
LRCC 5310	4.8	0.4	17.7	1,053

a–c Contents of impurities were evaluated for endotoxin, protein, and nucleic acid by using Limulus amoebocyte lysate assay, BCA protein assay, and optical density using spectrophotometer, respectively.

d Total weight of lyophilized LTA per total weight of bacterial pellet subjected to LTA isolation.

### Most Lp.LTAs Inhibit *E. faecalis* Biofilm Formation

**Figure 2:**
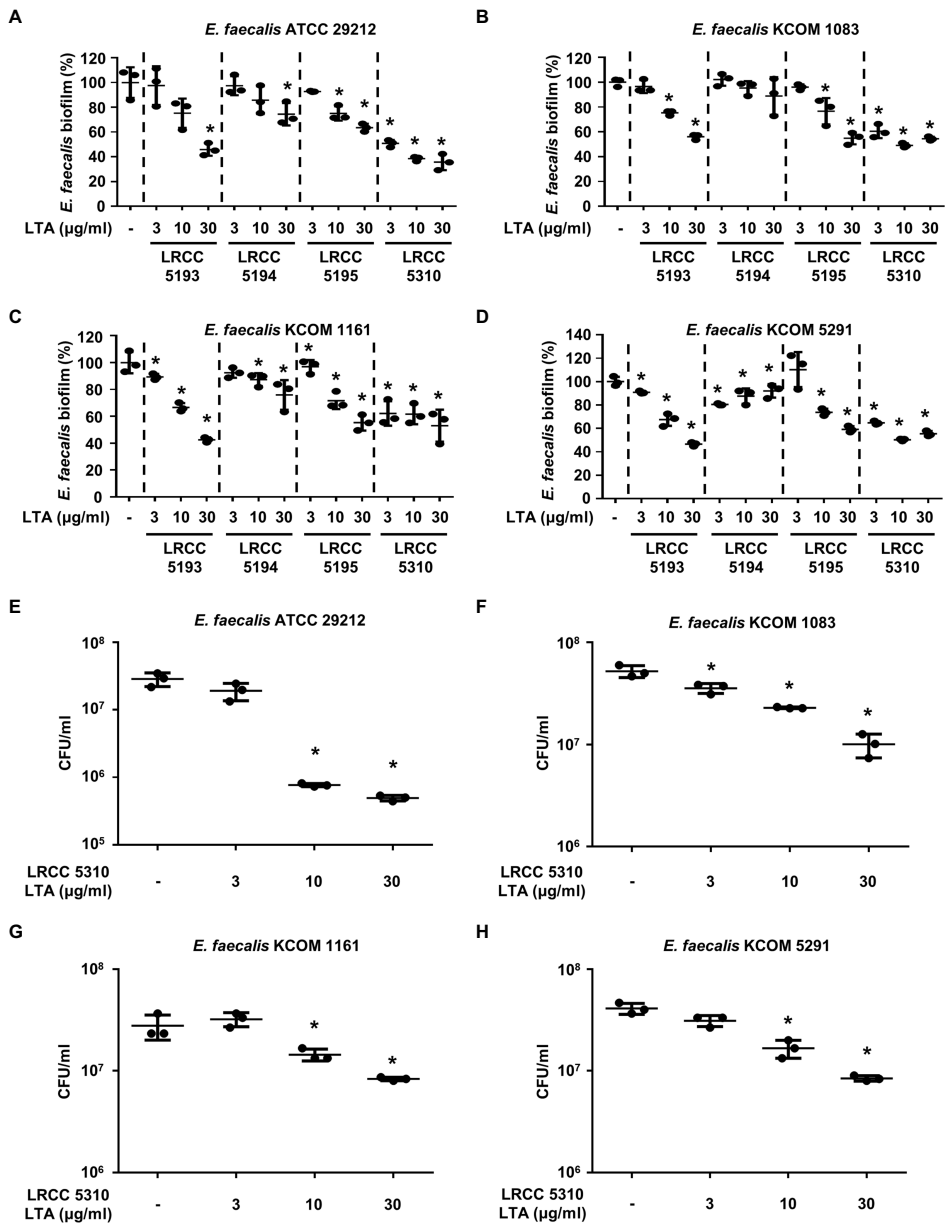
Lp.LTAs dose-dependently inhibit *E. faecalis* biofilm formation. Four strains of *E. faecalis*, including **(A,E)** a laboratory (ATCC 29212) and (**B–D**,**F–H**) three clinically isolated strains (KCOM 1083, KCOM 1161, and KCOM 5291), at 1×10^7^CFU/ml were grown on 96-well plates in the presence or absence of the indicated concentrations of **(A–D)** various Lp.LTA or **(E–H)** LRCC 5310 Lp.LTA at 37°C for 24h. After the incubation, biofilm formations were determined by crystal violet staining as described in Materials and Methods. Biofilm formation is presented as percentage change±standard deviation of triplicates against the non-treatment group set as 100%. **(E–H)** After collecting the biofilm, it was serially diluted and incubated on the THY agar plate at 37°C for 48h, and CFU formation was examined. Asterisk (*) indicates statistical significance at *p*<0.05 between the non-treatment and each Lp.LTA treatment groups.

We compared the anti-biofilm activity of the prepared Lp.LTAs against a total of four *E. faecalis* strains, including a laboratory strain (ATCC 29212) and three dental clinical isolates (KCOM 1083, KCOM 1161, and KCOM 5291) by crystal violet staining. As shown in Figures 2A–D, Lp.LTAs from LRCC 5193, 5195, and 5310 inhibited the biofilm formation of all *E. faecalis* strains in a dose-dependent manner and LRCC 5310 Lp.LTA most effectively inhibited biofilm formation of all strains of *E. faecalis* compared with other Lp.LTAs. However, LRCC 5194 Lp.LTA showed the anti-biofilm activity in a clinically isolated *E. faecalis* strain, KCOM 1161, but not in other strains. To determine whether the anti-biofilm activity of Lp.LTAs resulted from changes in bacterial number within the biofilm, we further examined the CFU formation of the biofilms cultured under the aforementioned condition. When four strains of *E. faecalis* biofilms were formed in the presence of various concentrations of LRCC 5310 Lp.LTA, CFU of *E. faecalis* biofilms were dose-dependently decreased by LRCC 5310 Lp.LTA, suggesting that the anti-biofilm activity of Lp.LTAs is mediated through the reduction of bacterial number (Figures 2E–H).

### Lp.LTAs Have Differential Anti-biofilm Activity on *S. gordonii* and *S. mutans*

**Figure 3:**
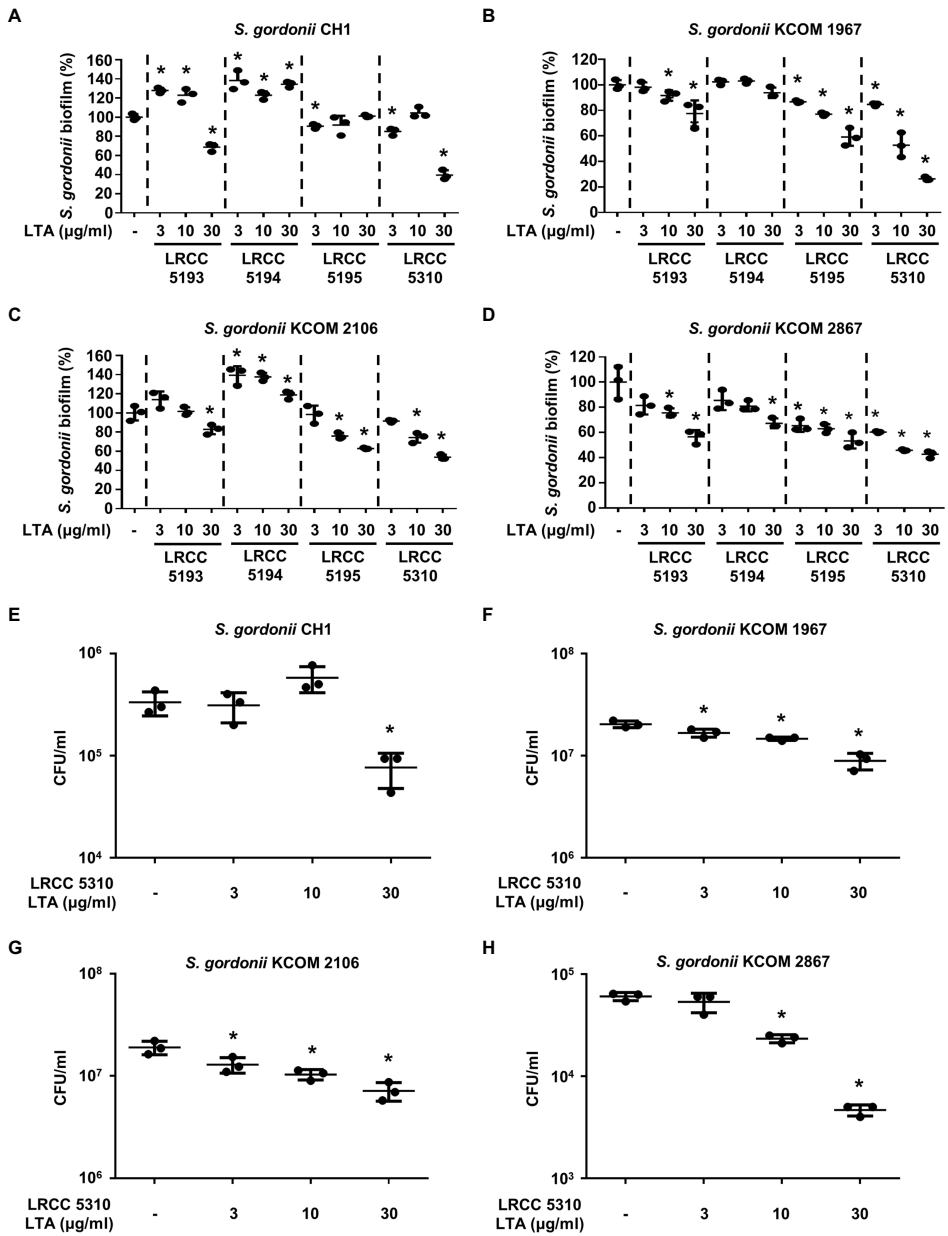
Lp.LTAs have different anti-biofilm activity on *S. gordonii*. Total of four strains of *S. gordonii,* including **(A,E)** a laboratory (CH1) and (**B–D**,**F–H**) three clinically isolated strains (KCOM 1967, KCOM 2106, and KCOM 2867), were grown on 96-well plates in the presence or absence of the indicated concentrations of **(A–D)** various Lp.LTA or **(E–H)** LRCC 5310 Lp.LTA at 37°C for 24h. After the incubation, biofilm formations were determined by crystal violet staining assay as described in Materials and Methods. Biofilm formation is presented as percentage change±standard deviation of triplicates against the non-treatment group set as 100%. **(E–H)** After collecting the biofilm, it was serially diluted and incubated on the THY agar plate at 37°C for 48h, and CFU formation was examined. Asterisk (*) indicates statistical significance at *p*<0.05 between the non-treatment and each Lp.LTA treatment groups.

**Figure 4:**
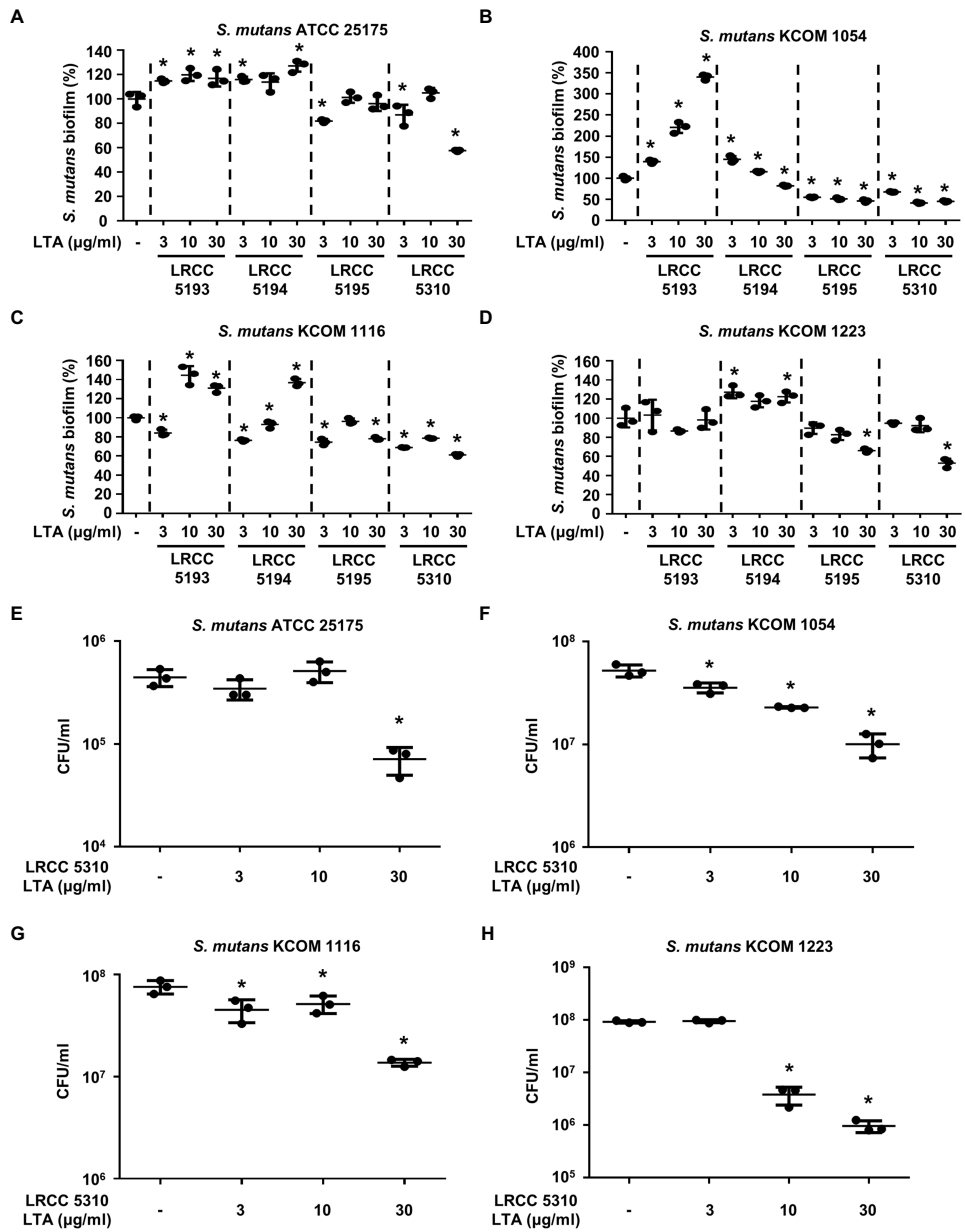
Lp.LTAs differently suppress *S. mutans* biofilm formation. Four different strains of *S. mutans*, including **(A,E)** a laboratory (ATCC 25175) and (**B–D**,**F–H**) three clinically isolated strains (KCOM 1054, KCOM 1116, and KCOM 1223), were grown on 96-well plates in the presence or absence of the indicated concentrations of **(A–D)** various Lp.LTA or **(E–H)** LRCC 5310 Lp.LTA at 37°C for 24h. After the incubation, biofilm formations were determined by crystal violet staining as described in Materials and Methods. Biofilm formation is presented as percentage change±standard deviation of triplicates against the non-treatment group set as 100%. **(E–H)** After collecting the biofilm, it was serially diluted and incubated on the THY agar plate at 37°C for 48h, and CFU formation was examined. Asterisk (*) indicates statistical significance at *p*<0.05 between the non-treatment and each Lp.LTA treatment groups.

To evaluate the anti-biofilm effects of Lp.LTAs on other dental pathogens, we also examined biofilm formation of *S. gordonii* and *S. mutans* in the presence of Lp.LTAs by crystal violet staining. When various concentrations of Lp.LTAs were treated to four different strains of *S. gordonii*, including a laboratory strain (CH1) and three dental clinical isolates (KCOM 1967, KCOM 2106, and KCOM 2867), LRCC 5193, 5195, and 5310 Lp.LTA dose-dependently reduced the biofilm formation of all *S. gordonii* strains (Figures 3A–D). Similar to the results from *E. faecalis*, LRCC 5310 Lp.LTA showed the most potent anti-biofilm activity among the tested Lp.LTAs, while LRCC 5194 Lp.LTA rarely affected or even enhanced biofilm formation of *S. gordonii*. Next, we also evaluated the effect of Lp.LTAs on *S. mutans* biofilm formation and found that the biofilm formations of four strains of *S. mutans* were effectively inhibited by 30μg/ml of LRCC 5310 Lp.LTA treatment (Figures 4A–D). Although lower concentrations of LRCC 5310 Lp.LTA (3 and 10μg/ml) inhibited the biofilm formation of *S. mutans* KCOM 1054, the inhibitory effect was marginal or not effective on the biofilm formation of other *S. mutans* strains. In addition, effects of other LRCC Lp.LTAs on *S. mutans* biofilm were variable depending on the concentrations. Of note, *S. mutans* biofilm was even enhanced by Lp.LTAs from LRCC 5193 (ATCC 25175, KCOM 1054, and KCOM 1116) and 5194 (ATCC 25175, KCOM 1054, KCOM 1116, and KCOM 1223). On the other hand, we also examined the CFU formation of *S. gordonii* and *S. mutans* biofilms cultured in the presence or absence of various concentrations of LRCC 5310 Lp.LTA. Similar to the results from CFU of *E. faecalis* biofilm, CFUs from both *S. gordonii* and *S. mutans* biofilm were dose-dependently attenuated by LRCC 5310 Lp.LTA (Figures 3E–H, 4E–H). Collectively, these results demonstrated that Lp.LTAs have differential anti-biofilm activity on *S. gordonii* and *S. mutans*. Furthermore, LRCC 5310 Lp.LTA was the most effective to control biofilm formation of dental pathogens and its anti-biofilm activity is caused by decrease in bacterial number within the biofilm.

### Lp.LTA Suppresses Biofilm Formation of Clinical Isolates of Dental Pathogenic Bacteria on Human Dentin Slices

**Figure 5:**
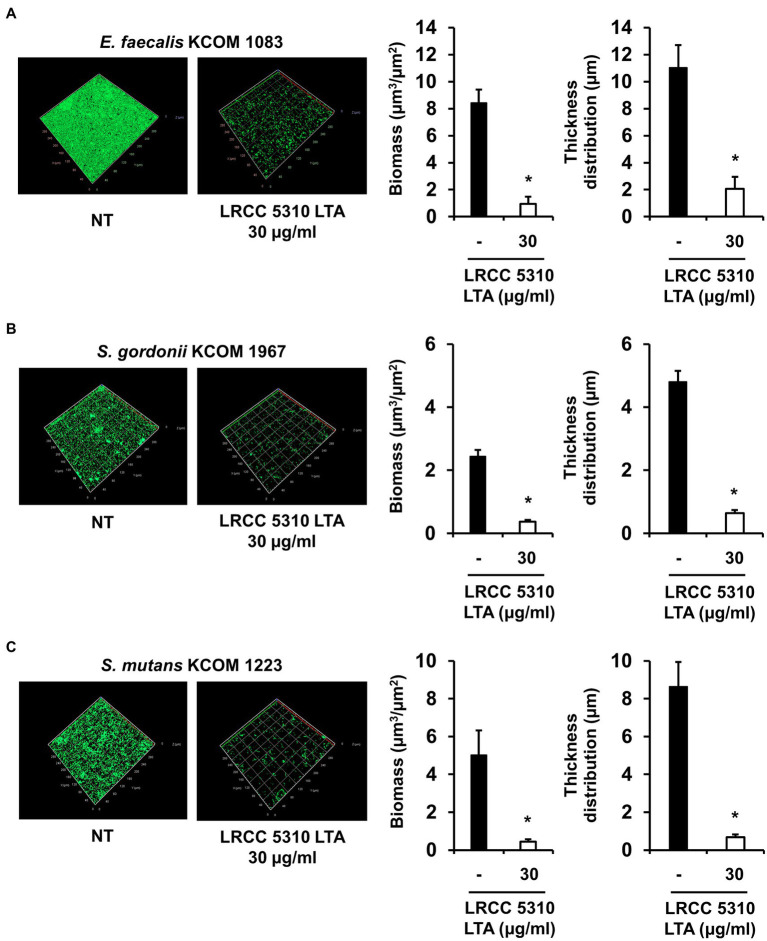
LRCC 5310 Lp.LTA inhibits biomass and thickness distribution of biofilm formed by clinically isolated *E. faecalis*, *S. gordonii*, and *S. mutans*. Clinically isolated strains of **(A)** *E. faecalis* (KCOM 1083), **(B)** *S. gordonii* (KCOM 1967), and **(C)** *S. mutans* (KCOM 1223) at 1×10^7^CFU/ml were grown on glass-bottom confocal dishes at 37°C for 24h in the presence or absence of 30μg/ml of LRCC 5310 Lp.LTA. After the incubation, biofilms were stained with the LIVE/DEAD BacLight Bacterial Viability Kit containing SYTO9 and propidium iodide and then visualized by confocal laser microscopy (green SYTO9; red, propidium iodide). The biomass and thickness distribution of biofilms were quantified using COMSTAT2 software. Asterisk (*) indicates statistical significance at *p*<0.05 between the non-treatment and LRCC 5310 Lp.LTA treatment groups.

**Figure 6:**
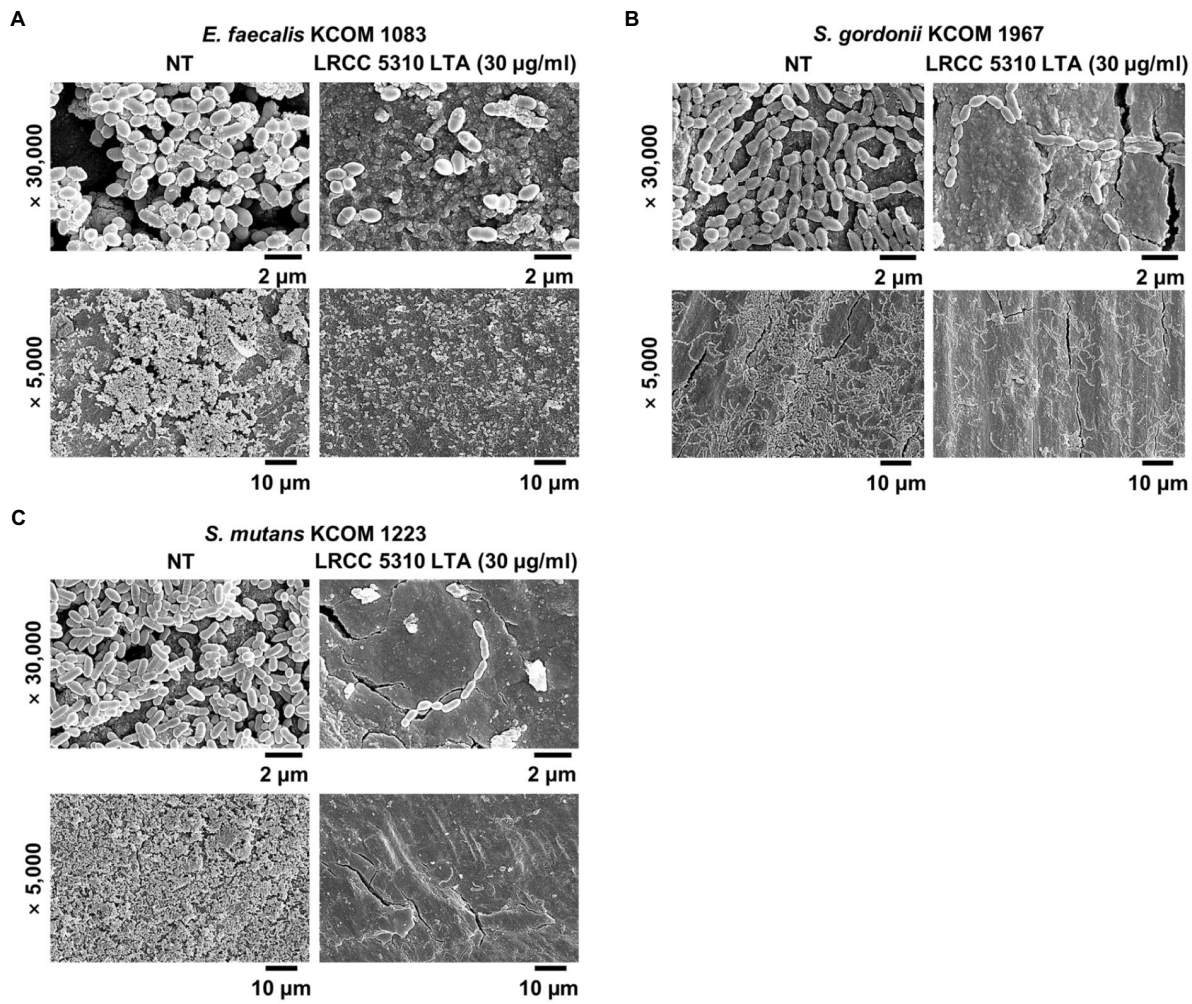
LRCC 5310 Lp.LTA suppresses biofilm formation of clinically isolated dental pathogens on human dentin slices. Clinically isolated strains of **(A)** *E. faecalis* (KCOM 1083), **(B)** *S. gordonii* (KCOM 1967), and **(C)** *S. mutans* (KCOM 1223) at 1×10^7^CFU/ml was grown on human dentin slices in the presence or absence of 30μg/ml of LRCC 5310 Lp.LTA at 37°C for 24h. Images were obtained by scanning electron microscopy with magnification at ×5,000 and ×30,000. Scale bars given in each image indicate 2 or 10μm.

The anti-biofilm activity of LRCC 5310 Lp.LTA, which showed the most effective anti-biofilm activity, was then confirmed by confocal laser scanning microscopic analysis against clinical isolates of *E. faecalis* (KCOM 1083), *S. gordonii* (KCOM 1967), and *S. mutans* (KCOM 1223). As shown in Figures 5A–C, the biomass and thickness distribution of all biofilms were significantly attenuated by 30μg/ml of LRCC 5310 Lp.LTA treatment. To evaluate the clinical application of LRCC 5310 Lp.LTA on *ex vivo* model, *E. faecalis* (KCOM 1083), *S. gordonii* (KCOM 1967), and *S. mutans* (KCOM 1223) were grown on human dentin slices in the presence or absence of 30μg/ml of LRCC 5310 Lp.LTA for 24h, and biofilm formations were then visualized by scanning electron microscope. As shown in Figures 6A–C, LRCC5310 Lp.LTA dramatically reduced the biofilm formation of clinical isolates of *E. faecalis*, *S. gordonii*, and *S. mutans*, suggesting that LRCC 5310 Lp.LTA can be used as an alternative anti-biofilm agent for controlling major Gram-positive dental pathogenic bacteria, that are closely related to pathogenicity of periodontitis, gingivitis, and dental caries.

## Discussion

In this study, we demonstrated that LRCC Lp.LTAs purified from four different strains of *L. plantarum* inhibited the biofilm formation of clinically isolated dental pathogens, including *E. faecalis*, *S. gordonii*, and *S. mutans* at *in vitro* level. Among the tested LRCC Lp.LTAs, LRCC 5310 Lp.LTA showed the most effective anti-biofilm activity against all strains of dental pathogens. Furthermore, the result from *ex vivo* experiments indicated that LRCC 5310 Lp.LTA effectively suppresses biofilm formation of dental pathogens on human dentin slices.

The differential anti-biofilm activities of various Lp.LTAs are supposedly linked to the diversity in their molecular structure. Lp.LTA is an amphiphilic molecule composed of hydrophilic poly-Gro-P backbone having D-alanine and/or hexose residues and a hydrophobic triacyl glycolipid anchor (Jang et al., 2011; Kang et al., 2016; Shiraishi et al., 2016). According to a previous study, the strain-specific diversity of Lp.LTA structure mainly diversified in the number of Gro-P backbone and D-alanine contents (Shiraishi et al., 2016). In fact, *L. plantarum* JCM 1149 LTA has 110 repeating units of Gro-P backbone with 42% D-alanine content, while *L. plantarum* L137 has 96 repeating units of Gro-P with 50% D-alanine content, suggesting that Lp.LTA has relatively lower D-alanine contents as length of Gro-P repeating units increased (Hatano et al., 2015). Furthermore, these structural differences would determine the biological potency of Lp.LTAs. For example, Lp.LTA having higher D-alanine contents is a more potent inducer of IL-12 than other Lp.LTA with relatively lower D-alanine contents (Hatano et al., 2015). Based on our results from phosphate assay and Western blot analysis, the isolated LRCC Lp.LTAs may have different numbers of Gro-P backbone, which determine their size, hydrophobicity, and net charge. Since phosphate of LRCC 5310 Lp.LTA was detected in relatively broader and later fractions during LTA isolation steps, LRCC 5310 Lp.LTA may have relatively higher number of Gro-P backbone with lower D-alanine contents leading to greater hydrophilicity compared with other LRCC Lp.LTAs. In addition, our results also suggest that LRCC 5310 Lp.LTA may have a relatively negative net charge because of its higher number of negatively charged phosphate residues within Gro-P backbone than other LRCC Lp.LTAs. Although further study is needed to confirm the correlation between differential anti-biofilm activity and LTA structure, these results suggested that the differential anti-biofilm activity of Lp.LTAs could correlate with their molecular structure variation in length of Gro-P repeating units and D-alanine content.

In the present study, although Lp.LTAs from LRCC 5193 and 5194 showed anti-biofilm activity for most *E. faecalis* and *S. gordonii* strains, they did not affect or even enhanced the biofilm formation of *S. mutans* strains. According to our previous study, anti-biofilm activity of Lp.LTA on *S*. *mutans* can be achieved by several proposed mechanisms. First, Lp.LTA can compete and interfere with the binding of *S. mutans* to surface materials subsequently leading to inhibition of its biofilm formation (Ahn et al., 2018a). Second, Lp.LTA can suppress sucrose decomposition which blocks glucan production needed for EPS formation (Ahn et al., 2018a). Based on the structural variation of Lp.LTAs, Lp.LTAs from LRCC 5193 and 5194 may exert weaker anti-biofilm activity against *S. mutans* than LRCC 5310 Lp.LTA through the aforementioned mechanisms. On the other hand, enhanced biofilm formation of several *S. mutans* strains by Lp.LTAs from LRCC 5193 and 5194 can be explained by several possible mechanisms. As previously mentioned, we speculated that Lp.LTAs from LRCC 5193 or 5194 may have relatively higher D-alanine contents compared with LRCC 5310 Lp.LTA, and these abundant D-alanine moieties may play a role in biofilm formation of *S. mutans*. Accumulated studies demonstrated that exogenous D-alanine increased biofilm formation of *S. mutans*. In fact, exogenous D-alanine restored the suppressed *S. mutans* biofilm formation by D-cycloserine, a competitive inhibitor of alanine racemase needed for decomposition of D-alanine (Qiu et al., 2016). Furthermore, it has also been reported that exogenous D-alanine enhanced the biofilm formation of *S. mutans* by promoting the expression of glucosyltransferases (GTFs), which induce EPS production by creating α-1,3- and α-1,6-linked glucan chains using sucrose and glucose (Liu et al., 2018). However, it is still unclear whether LRCC 5193 and 5194 Lp.LTA regulate the expression or activities of GTFs in *S. mutans*.

In the current study, LRCC 5310 Lp.LTA dose-dependently reduced the CFU formation within biofilm of dental pathogens, suggesting that anti-biofilm activity of LRCC Lp.LTAs is mediated through reduction in bacterial number in the biofilm. Although further studies are needed, reduced bacterial number by LRCC Lp.LTAs may be explained by some possible mechanisms. First, LRCC Lp.LTAs could inhibit initial attachment of bacteria by direct interaction with bacteria or regulating quorum-sensing signaling. In our previous studies, inhibitory effect of Lp.LTA on *E. faecalis*, *S. mutans*, and *S. aureus* biofilm formation initiated at early time point (less than 3h; Ahn et al., 2018a,b; Jung et al., 2019). Moreover, surface attachment of *S. mutans* was effectively blocked on Lp.LTA pre-coated plate (Ahn et al., 2018a). Therefore, LRCC Lp.LTAs may suppress bacterial attachment by its direct interaction with bacteria. Second, enhanced autoinducer-2 (AI-2) quorum-sensing molecule by Lp.LTA suppressed *S. aureus* biofilm formation by downregulating intracellular adhesion (*ica*) gene needed for production of polysaccharide intercellular adhesion (Ahn et al., 2018b). Since *E. faecalis*, *S. gordonii*, and *S. mutans* commonly share the AI-2-mediated quorum-sensing system, regulation of quorum-sensing signaling by LRCC Lp.LTAs might be another potential mechanism. Third, LRCC Lp.LTAs could promote the dispersion of dental pathogens from the biofilm by upregulating protease activity. In fact, LapG and Spl protease secreted from *Pseudomonas putida* and *S. aureus* evoke the dispersion of bacteria from their biofilm (Kaplan, 2010). Furthermore, since disruptive property of Lp.LTA on pre-formed biofilms of *E. faecalis*, and *S. aureus* was observed in our previous studies (Ahn et al., 2018b; Jung et al., 2019), facilitated bacterial dispersion by LRCC Lp.LTAs might be another potential mechanism responsible for the reduced bacterial number, subsequently leading to decrease in biofilm formation of dental pathogens.

Since biofilms of dental pathogens are difficult to be eradicated by antibiotics and disinfectants, an effective alternative strategy for controlling biofilm formation of dental pathogens is needed. For this reason, we isolated LTAs from four different strains of *L. plantarum* and tested their anti-biofilm activity against major Gram-positive dental pathogens including *E. faecalis*, *S. gordonii*, and *S. mutans*. Although all of the tested LRCC Lp.LTAs inhibited biofilm formation of laboratory and clinically isolated strains of the dental pathogens, LRCC 5310 Lp.LTA had the most effective and broadest anti-biofilm activity. Although anti-biofilm efficacy of LRCC 5310 Lp.LTA should be confirmed in pre-clinical and clinical test models, our findings provide evidence that LRCC 5310 Lp.LTA could be applied as a useful anti-biofilm agent to prevent biofilm formation of *E. faecalis*, *S. gordonii*, and *S. mutans*. In addition, although anti-biofilm activity of LRCC 5310 Lp.LTA was similar to that of LTA from a laboratory strain of *L. plantarum* KCTC10887BP (KCTC10887BP Lp.LTA) against the biofilm formation of *E. faecalis* ATCC 29212 (Kim et al., 2020), a total amount of LTA recovered from LRCC 5310 strain was remarkably higher than from KCTC10887BP strain (1,053μg/g bacterial pellet of LRCC 5310 vs. 568μg/g bacterial pellet of KCTC10887BP). Thus, LRCC 5310 Lp.LTA might be cost-effective and more beneficial for its clinical application than KCTC10887BP Lp.LTA. Moreover, since we also previously reported that pretreatment of Lp.LTA effectively cooperated with dental disinfectants, such as chlorhexidine digluconate and calcium hydroxide, for disrupting preformed biofilm of various dental pathogens (Kim et al., 2019, 2020), LRCC 5310 Lp.LTA may also be used as a supplementary medicament to treat dental diseases such as apical periodontitis and dental caries.

## Data Availability Statement

The data presented in this study are deposited in GenBank repository, Accession Number: LRCC 5193 (OK493617); LRCC 5194 (OK493618); LRCC 5195 (OK493619); LRCC 5310 (OK493620).

## Ethics Statement

The studies involving human participants were reviewed and approved by Institutional Review Board of Seoul National University Dental Hospital, Seoul, Republic of Korea (CRI 17010). The patients/participants provided their written informed consent to participate in this study.

## Author Contributions

DL and SH designed the research. DL, JI, DP, MP, and SY carried out the experiments. DL, JI, DP, SJ, JP, and SH analyzed and interpreted the data. DL, JI, SJ, and SH prepared and reviewed the manuscript. All authors contributed to the article and approved the submitted version.

## Funding

This work was supported by grants from the Lotte Confectionary Co. Ltd. and the National Research Foundation (NRF) funded by the Korean government (NRF-2018R1A5A2024418, NRF-2019R1A2C2007041, and 2020M3H1A1073304). The funders were not involved in the study design, collection, analysis, interpretation of data, the writing of this article, or the decision to submit it for publication.

## Conflict of Interest

MP, SY, and JP are employed by Lotte R&D Center.

The remaining authors declare that the research was conducted in the absence of any commercial or financial relationships that could be construed as a potential conflict of interest.

## Publisher’s Note

All claims expressed in this article are solely those of the authors and do not necessarily represent those of their affiliated organizations, or those of the publisher, the editors and the reviewers. Any product that may be evaluated in this article, or claim that may be made by its manufacturer, is not guaranteed or endorsed by the publisher.
